# Anti-biofilm and remineralization effects of chitosan hydrogel containing amelogenin-derived peptide on initial caries lesions

**DOI:** 10.1093/rb/rby005

**Published:** 2018-03-21

**Authors:** Qian Ren, Zhongcheng Li, Longjiang Ding, Xiuqing Wang, Yumei Niu, Xi Qin, Xuedong Zhou, Linglin Zhang

**Affiliations:** State Key Laboratory of Oral Diseases; National Clinical Research Center for Oral Diseases; and Department of Cariology and Endodonics, West China Hospital of Stomatology, Sichuan University, No. 14, 3rd Section, Renmin Nan Lu, Chengdu, People’s Republic of China

**Keywords:** caries, amelogenin-derived peptide, chitosan, remineralization

## Abstract

In this study, we have designed a more clinically powerful anti-caries treatment by applying the amelogenin-derived peptide QP5 to the antibacterial carrier material chitosan in a hydrogel (CS-QP5 hydrogel), and characterized its effects on the inhibition of a cariogenic biofilm and the promotion of the remineralization of the initial caries lesions. The results indicated that the CS-QP5 hydrogel sustainably inhibited the growth of the *Streptococcus mutans* biofilm, lactic acid production and the metabolic activity over a prolonged period of time. Moreover, the CS-QP5 hydrogel promoted the remineralization of early enamel lesions, which were indicated by surface micro-hardness (, polarized light microscopy and transverse microradiography. In conclusion, the CS-QP5 hydrogel shows good potential for caries control in the clinic because of its antibacterial effects as well as the remineralization of initial enamel carious lesions even in a biofilm model over a prolonged period of time.

## Introduction

Dental caries is a dietary carbohydrate-modified bacterial infectious disease and is one of the most common global oral health problems in the world today [1]. It is defined as the destruction of the dental hard acellular tissue by acidic by-products from the bacterial fermentation of dietary carbohydrates, particularly sucrose. It progresses slowly in most of the people and is attributed to an ecological imbalance in the equilibrium between tooth minerals and oral biofilms, which is characterized by microbial activity, resulting in fluctuations in the plaque pH due to bacterial acid production, suffering action from the saliva and the surrounding tooth structure [2]. The microbial community of caries is diverse, and *Streptococcus mutans* is most primarily associated with it [3, 4]. According to the pathological mechanism due to which caries occurs, the control of pathogenic bacteria and the inhibition of demineralization and promotion of remineralization are two critical aspects of the progress of caries prevention [5, 6]. Numerous clinical and laboratory studies have demonstrated the anti-caries effect of fluoride, which can shift the demineralization/remineralization balance by cariostatic efficacy, inhibition of demineralization, promotion of incipient lesion remineralization [7, 8]. However, fluoride has the potential to cause fluorosis while overused, and some fluoride product like silver diamine fluoride may cause black staining of the carious lesion [9–11]. Therefore, the alternative, effective no-fluoride anti-caries agents need to be explored.

Excellent progress has been made in the recent years in the promotion of remineralization or inhibition of the demineralization of caries lesions through biomimetic mineralization triggered by leucine-rich amelogenin peptides [12, 13], multiple-DSS peptides derived from dentin phosphoprotein [14, 15], peptides with beta-sheet structures [16] and self-assembling peptide-amphiphiles [17]. In the previous studies, our group also confirmed the remineralization potential of amelogenin and its derived peptides, which were designed and synthesized independently. First, Xiang et al. [18] found that the enamel matrix derivative plays an essential role in promoting the remineralization of the initial enamel carious lesions. Then, based on the highly conserved Gln-Pro-X repeats in amelogenin, which serve as a biomimetic scaffold for nucleating hydroxyapatite and promoting mineralization, our group designed a novel peptide containing three Gln-Pro-X repeats (QPHQPMQPQ) and a sequence with a high hydroxyapatite binding capacity (SVSVGMKPSPRP). We demonstrated the peptide’s remineralization ability *in vitro* [19]. To promote nucleation as well as render the synthetic peptide highly soluble and stable in water, we added a seven-residue hydrophilic segment (TKREEVD) to a sequence of five Gln-Pro-X repeats (QPYQPVQPHQPMQPQ) and produced another amelogenin-derived peptide, called QP5. As reported in previous studies, it can significantly promote the remineralization of the initial caries in the bovine enamel and the rat enamel without any toxic effects [20, 21]. However, it has no effects on limiting the growth of cariogenic bacteria, which is an important contributor to the caries onset and progression. In addition, the use of this peptide in clinical practice is limited, as its residence time with effective concentration on the tooth surface is relatively short when applied in the form of an aqueous solution. Thus, we need to urgently find an antibacterial carrier to achieve the goal of the simultaneous control of bacteria and promotion of the remineralization during the progress of caries prevention.

Chitosan is a biocompatible and non-toxicity polymer obtained by the deacetylation of chitin, one of the most common polymers in nature, and has been widely used in drug delivery systems and tissue engineering [22, 23]. Its positive charge accumulates on the cell walls of the bacteria, promoting a bactericidal and bacteriostatic property coupled with the ability to form a film and adhere to the tooth, making chitosan an ideal base for sustained drug release [24]. Previous studies have shown that chitosan can effectively inhibit *S. mutans* adherence and biofilm formation, exhibiting a significant antibacterial and plaque reducing action [25, 26]. Because of its interesting properties, chitosan has been widely used in various long-lasting oral formulations; it exhibits a synergistic antiplaque effect when it associated with propolis, nano-silver and chlorhexidine [27–29]. In recent studies, chitosan has also been used in formulations, such as toothpastes (Chitodent), mouthwash solutions and chewing gums, for clinical caries control [30–33]. In all its forms, chitosan shows the antibacterial activity of the *Streptococcus* bacteria groups, inhibits the growth and adherence of cariogenic bacteria and the demineralization process of the dental enamel *in vitro* and stimulates salivation *in vivo*.

Dental plaque is one of the most necessary factors for caries, and it plays a significant role in the dynamic equilibrium of the mineralization and the demineralization on the tooth surface [3]. When the pH falls below a critical value, demineralization occurs, while an increase in the mineral content (remineralization) occurs with an increase in the pH. In this study, our goal was to (i) apply the amelogenin-derived peptide QP5 to antibacterial carrier chitosan to obtain a dual anti-caries complex, (ii) determine the long-term effects of a chitosan hydrogel containing QP5 (CS-QP5 hydrogel) on an *S. mutans* biofilm and (iii) determine whether the CS-QP5 hydrogel can promote the remineralization of the early enamel lesions in the biofilm model. To the best of our knowledge, this the first study on the antibacterial and remineralization effects of a CS-QP5 hydrogel in a biofilm model. The CS-QP5 hydrogel may present good potential for caries control in clinical practice.

## Materials and methods

### CS-QP5 hydrogel preparation

QP5 was synthesized by GL Biochem (Shanghai, China) using standard Fmoc solid-phase chemistry on an Apex 396 multiple peptide synthesizer (AAPPTec, Louisville, KY, USA). The peptide sequence and integrity were confirmed using electrospray ionization mass spectrometry (Shimadzu, Kyoto, Japan) and reverse-phase high-performance liquid chromatography (RP-HPLC; CHTH Sci. and Tech, Beijing, China). Peptide purity was determined to be >95.0% by using RP-HPLC. Chitosan of medium molecular weight (75–85% deacetylated) was purchased from Sigma-Aldrich (MI, USA).

The CS-QP5 hydrogel was prepared using the protocol developed by Ruan et al. [34] with some modifications. In total 1 ml of a chitosan calcium phosphate solution (960 µl of 1% chitosan, 25 µl of 0.1 M CaCl_2_ and 15 µl of 0.1 M Na_2_HPO_4_) was mixed with QP5 to obtain the final concentration of 25 µM, followed by stirring overnight at room temperature. The pH value was adjusted to 6.0 by using dilute NaOH.

### Specimen preparation

Bovine permanent incisors free of lesions, cracks and fluoric mottle were selected; crowns were separated from the roots and cut into sections measuring ∼5 × 5 × 2 mm by using a diamond-coated band saw with continuous water cooling (Struers Minitom; Struers, Copenhagen, Denmark). Enamel blocks were embedded in polymethylmethacrylate and painted with two layers of acid-resistant nail varnish, leaving a 4 × 4 mm window exposed on the labial enamel surface. These surfaces were then ground flat with water-cooled carborundum discs of waterproof silicon carbide paper (Struers) of various grits (1000, 1200, 2400, 3000 and 4000). All of the polished samples were individually sonicated in distilled water for 5 min to remove the residual abrasives.

Before the formation of the caries lesions, the baseline surface micro-hardness (SMH_0_) of the enamel blocks was measured. Five indentations at intervals of 100 μm were made at the center of each enamel block surface. The measurements were performed with a micro-hardness tester (Duramin-1/-2, Struers) and a Knoop indenter at a load of 50 g for 15 s. Enamel blocks with SMH_0_ = 330–400 Knoop hardness number were selected for further study.

The initial enamel caries lesions were produced in enamel blocks as described earlier in [20, 35, 36]. The demineralization solution contained 50 mM acetic acid (pH 4.5), 2.2 mM Ca(NO_3_)_2_, 2.2 mM KH_2_PO_4_, 5.0 mM NaN_3_ and 0.5 ppm NaF. The blocks were immersed in the demineralization solution (8 ml per block) at 37°C for 3 days under continuous, low-speed magnetic stirring (100 rpm). The post-demineralization surface micro-hardness (SMH_1_) was measured as described after introducing five new indentations located at least 100 mm from the ones used to measure SMH_0_. In all, 90 enamel blocks with SMH_1_ = 140–180 Knoop hardness number were selected for further testing. Before the start of the experiment, all of the enamel blocks were sterilized with ultraviolet light for 4 h.

### Inoculation

The enamel blocks were inoculated as described earlier with some modification [37]. In brief, each enamel block was placed into a well of a 24-well plate with the enamel surface window facing up. In all, 90 enamel blocks were divided into 5 sets and treated with 1% chitosan hydrogel containing amelogenin-derived peptide (CS-QP5), 1% chitosan (CS), 25 μM amelogenin-derived peptide (QP5), 1000 ppm Fluoride (NaF) and distilled and deionized water (DDW) for 5 min before inoculation. Then, each specimen was transferred into a new 24-well plate with a 2-ml culture of *S. mutans* with 1% sucrose and incubated in 5% CO_2_ at 37°C. After 8 h, each specimen was transferred into a new 24-well plate with 2 ml of a fresh medium (3.7 g/l BHI medium containing 0.9 mM phosphate, 1.5 mM CaCl_2_ and 25 mM PIPES buffer, pH 7.0), and incubated in 5% CO_2_ at 37°C for 7 days.

### Colony-forming unit counts of adherent biofilms

Enamel blocks with 1-, 4- and 7-day biofilms were transferred into tubes with 2 ml of cysteine peptone water (CPW), and the biofilms were harvested by sonication and vortexing via a vortex mixer (Fisher, Pittsburgh, PA, USA). The bacterial suspensions were serially diluted and spread onto BHI agar plates, incubated anaerobically at 37°C for 48 h [38].

### Lactic acid production by adherent biofilms

Enamel blocks with 1-, 4- and 7-day biofilms (*n* = 3) were rinsed with CPW to remove loose bacteria and then, transferred to 24-well plates containing 1.5 ml of buffered-peptone water (BPW) plus 0.2% sucrose. The specimens were incubated for 3 h to allow the biofilms to produce acid and then rinsed with PBS for the next 3-[4, 5-dimethylthiazol-2-yl]-2, 5-diphenyltetrazolium bromide (MTT) assay. The BPW solutions were collected for the lactate analysis by using an enzymatic method. The 340-nm absorbance of BPW was measured with a microplate reader. Standard curves were prepared using a standard lactic acid solution (Supelco Analytical, Bellefonte, PA, USA) [39].

### MTT assay of biofilm metabolic activity

MTT assay was performed to examine the metabolic activity of the biofilms. The MTT assay is a colorimetric assay that measures the enzymatic reduction of MTT, a yellow tetrazole, to formazan. Enamel blocks with 1-, 4- and 7-day biofilms (*n* = 3) were transferred to a new 24-well plate, and 1 ml of the MTT dye (0.5 mg/ml MTT in PBS) was added to each well and incubated at 37°C in 5% CO_2_ for 1 h. During the incubation, metabolically active bacteria metabolized the MTT, a yellow tetrazole, and reduced it to purple formazan inside the living cells. The discs were then transferred to new 24-well plates, and 1 ml of dimethyl sulfoxide (DMSO) was added to solubilize the formazan crystals. The plates were incubated for 20 min with gentle mixing at room temperature. Next, 200 µl of the DMSO solution was collected, and its absorbance at 540 nm was measured via a microplate reader (SpectraMax M5, Molecular Devices, Sunnyvale, CA, USA). A higher absorbance value implied a higher formazan concentration, which indicated more metabolic activity in the biofilms [40].

### Surface micro-hardness analysis

The surface micro-hardness (SMH2) of the enamel blocks was measured after incubation for 1, 4 and 7 days, by using five new indentations located at least 100 μm from those used to measure SMH0 and SMH1. The mean SMH measurements after the demineralization determined at the baseline, after the inoculation were used to calculate the recovery (surface micro-hardness recovery [SMHR]) [41].

$$\text{SMHR}={\text{SMH}}_{2}-{\text{SMH}}_{1}$$

### Polarized light microscopy

Approximately 300-μm thick sections were cut vertically to the windows on the enamel surfaces by using a diamond-coated band saw (Struers). Then, all of the sections were polished to a thickness of ∼100 μm, which was verified using a digital micrometer (Mitutoyo, Tokyo, Japan). The slices were placed on glass microscope slides, immersed in DDW, and examined with a polarized light microscope (ECLIPSE ME600L, Nikon, Tokyo, Japan).

### Transverse microradiography

Each slice was fixed on the Plexiglass slides in a transverse microradiography (TMR) sample holder (Inspektor Research Systems BV, Amsterdam, The Netherlands). Then, the slices were microradiographed alongside an aluminum calibration step-wedge with a monochromatic CuK X-ray source (Philips, Eindhoven, The Netherlands) operated at 20 kV and 20 mA and an exposure time of 25 s. The lesion depth, mineral loss, and mineral content at the selected depths were analyzed with imaging software (Transversal Microradiography Software 2006, Inspektor Research Systems). Ten TMR traces were measured on each slice: five traces within areas not exposed to pH cycling and five within areas exposed to the cycling. Further, 10 slices were analyzed from each enamel block. The lesion depth was determined as the distance from the enamel surface to the point at which the mineral content reached 87% of that of sound enamel.

### Statistical analysis

The commercial software GraphPad Prism 7.0 for MAC (GraphPad Software Inc.) was used for carrying out the statistical analyses and graphics. The inter-group differences were assessed for significance using ANOVA, while the intra-group differences were assessed using Tukey’s HSD tests. The differences between groups were considered significant at *P* < 0.05.

## Results

**Figure 1.**
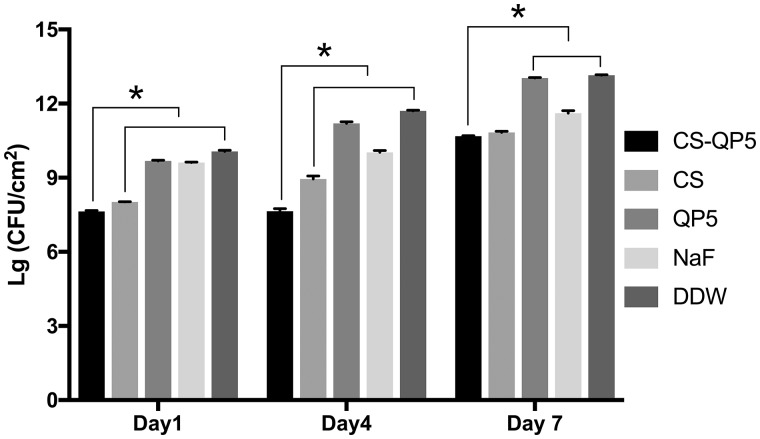
CFU counts for 1-, 4- and 7-day species treated with1% chitosan hydrogel containing amelogenin-derived peptide (CS-QP5), 1% chitosan (CS), 25 μM amelogenin-derived peptide (QP5), 1000 ppm fluoride (NaF) and DDW. Data are presented as mean ± SD. *P < 0.05

Figure 1 shows the colony-forming unit (CFU) counts for 1-, 4- and 7-day species treated with the CS-QP5 hydrogel, chitosan, QP5, NaF and DDW. On each day, treatment with the CS-QP5 hydrogel decreased the CFU, as compared with the DDW control. Although the CFU decreased in the NaF group, it was still higher than that in the CS-QP5 hydrogel group. With an increase in the inoculation time, the CFU of all of the groups increased, but the CFU of the CS-QP5 hydrogel and the chitosan groups was still less than that of the other groups. As expected, almost no antibacterial effect was observed in the samples treated with QP5.

**Figure 2.**
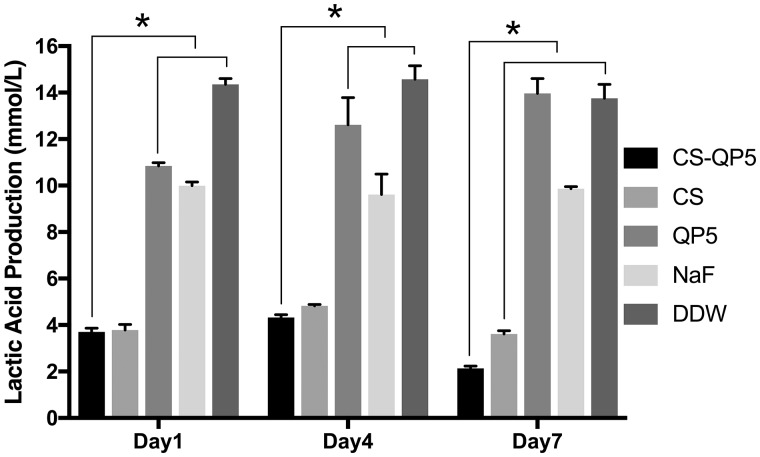
Lactic acid production for the 1-, 4-, and 7-day species treated with 1% chitosan hydrogel containing amelogenin-derived peptide (CS-QP5), 1% chitosan (CS), 25 μM amelogenin-derived peptide (QP5), 1000 ppm fluoride (NaF) and DDW. Data are presented as mean ± SD. *P < 0.05

Figure 2 shows the lactic acid production for the 1-, 4- and 7-day species treated with the CS-QP5 hydrogel, chitosan, QP5, NaF and DDW. The biofilms on the DDW control produced the maximum amounts of the acids. On each day, treatment with the CS-QP5 hydrogel or chitosan decreased the acid production, as compared with that of the NaF group. When the inoculation time was increased, the acid production of the NaF and DDW groups did not change considerably, but that of the QP5 group increased. Interestingly, the acid production of the CS-QP5 hydrogel and chitosan groups, particularly, the CS-QP5 hydrogel group, decreased obviously.

**Figure 3.**
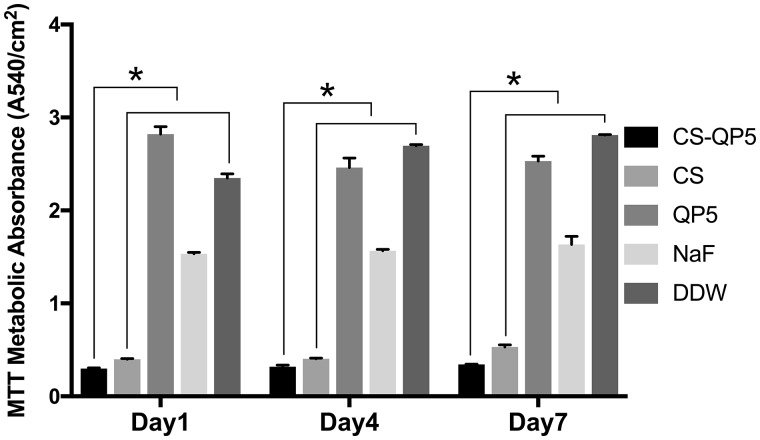
The biofilm metabolic activity of the 1-, 4- and 7-day species treated with 1% chitosan hydrogel containing amelogenin-derived peptide (CS-QP5), 1% chitosan (CS), 25 μM amelogenin-derived peptide (QP5), 1000 ppm fluoride (NaF) and DDW. Data are presented as mean ± SD. *P < 0.05

The biofilm metabolic activity of the 1-, 4- and 7-day species treated with the CS-QP5 hydrogel, chitosan, QP5, NaF and DDW was shown in Fig. 3. The MTT results showed that the biofilms treated with DDW had a high metabolic activity. On each day, treatment with the CS-QP5 hydrogel decreased the metabolic activity, as compared with the DDW control. Although the metabolic activity decreased in the NaF group, it was still higher than that in the CS-QP5 hydrogel group. When the inoculation time increased, the metabolic activity of all of the groups increased, but the CFU of the CS-QP5 hydrogel group was still less than that of the other groups. As expected, almost no effect was observed on the biofilm metabolic activity treated with QP5.

**Figure 4.**
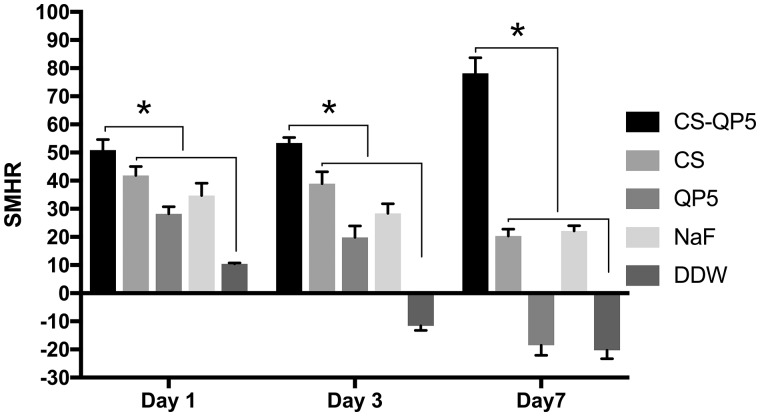
SMHR 1-, 4- and 7-day species treated with 1% chitosan hydrogel containing amelogenin-derived peptide (CS-QP5), 1% chitosan (CS), 25 μM amelogenin-derived peptide (QP5), 1000 ppm fluoride (NaF) and DDW. Data are presented as mean ± SD. *P < 0.05

A comparison of the SMH after the inoculation showed that the SMH values were significantly higher in the blocks treated with the CS-QP5 hydrogel on Days 1, 4 and 7, as shown in Fig. 4. On Day 1, all of the groups showed a growth trend of SMH, particularly the CS-QP5 hydrogel group. With an increase in the inoculation time, the SMH of all of the groups started to decrease except that of the CS-QP5 hydrogel. The DDW group showed a negative growth of the surface hardness from Day 4, while the QP5 group did so from Day 7. Although the chitosan and the NaF groups showed a decreasing trend, the SMH was still higher than that observed after the demineralization.

**Figure 5.**
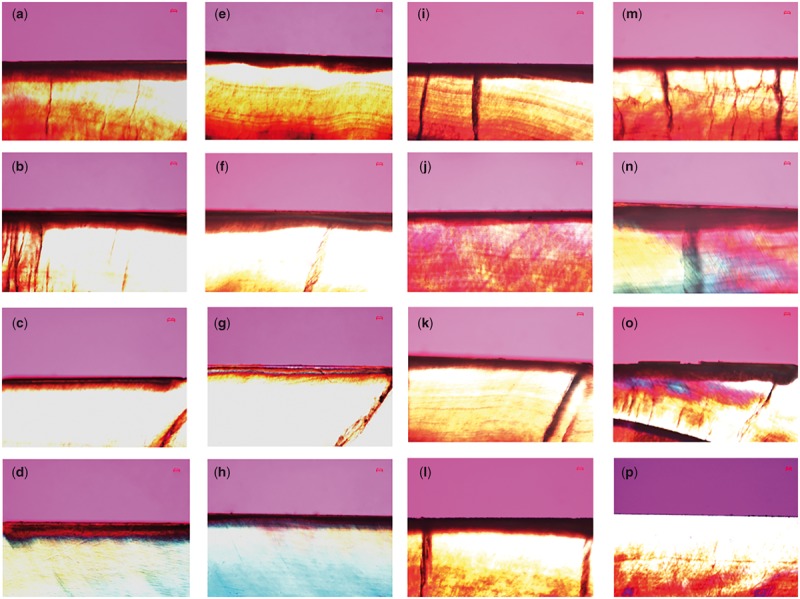
Polarized light micrographs of enamel sections after 1-, 4- and 7-day inoculation in the presence of (**a–c**) 1% chitosan hydrogel containing amelogenin-derived peptide (CS-QP5), (**d–f**) 1% chitosan (CS), (**g–i**) 25 μM amelogenin-derived peptide (QP5), (**j–l**) 1000 ppm fluoride (NaF) and (**m–o**) DDW. Picture (**p**) is the photo of a control group, with no demineralizing treatment. Scale Bar, 50 μm

In polarized light microscopy (PLM), the enamel surface layer exhibited negative birefringence and a relatively high mineral content because of the remineralization, while the lesion body under the surface layer exhibited positive birefringence because of its relatively low mineral content. As shown in Fig. 5, after inoculation for 1 day, a surface layer was clearly visible on the enamel treated with the CS-QP5 hydrogel. With an increase in the inoculation time, the lesions became significantly shallower and the negative birefringence of the enamel surface layer became more obvious in the CS-QP5 hydrogel group than in the other four groups. These results provided a direct evidence of the remineralization promoted by the CS-QP5 hydrogel.

**Figure 6.**
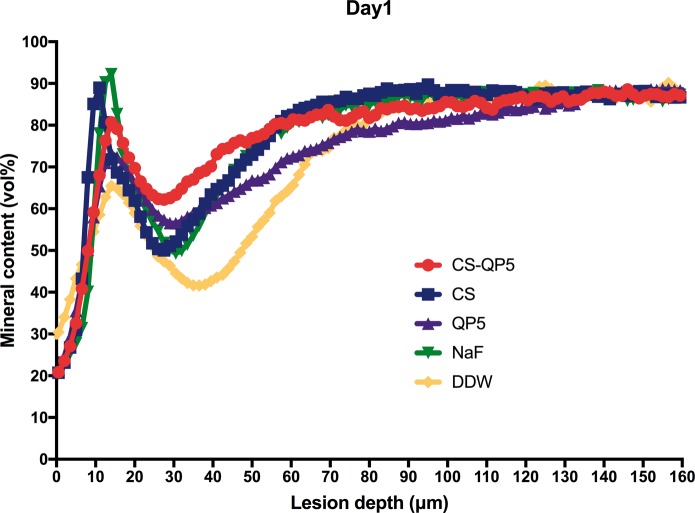
Mineral content at different depths after inoculation for 1 day in the presence of 1% chitosan hydrogel containing amelogenin-derived peptide (CS-QP5), 1% chitosan (CS), 25 μM amelogenin-derived peptide (QP5), 1000 ppm fluoride (NaF), and DDW. Profiles are averages (± SD) per experimental group (for each group, *n* = 10, five scans per specimen)

**Figure 7.**
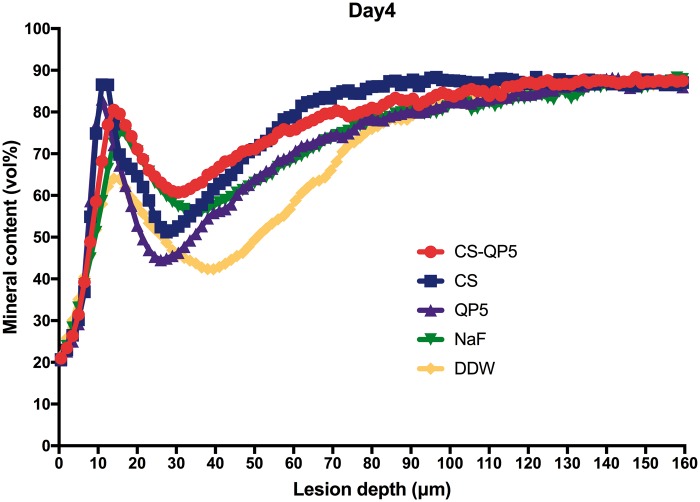
Mineral content at different depths after inoculation for 4 days in the presence of 1% chitosan hydrogel containing amelogenin-derived peptide (CS-QP5), 1% chitosan (CS), 25 μM amelogenin-derived peptide (QP5), 1000 ppm fluoride (NaF) and DDW. Profiles are averages (± SD) per experimental group (for each group, *n* = 10, five scans per specimen)

**Figure 8.**
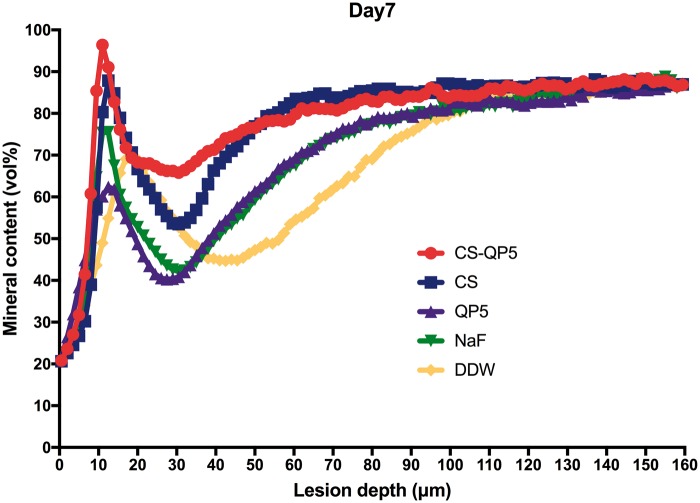
Mineral content at different depths after inoculation for 7 days in the presence of 1% chitosan hydrogel containing amelogenin-derived peptide (CS-QP5), 1% chitosan (CS), 25 μM amelogenin-derived peptide (QP5), 1000 ppm fluoride (NaF) and DDW. Profiles are averages (± SD) per experimental group (for each group, *n* = 10, five scans per specimen)

Figures 6–8 show the mineral content at different depths after inoculation for 1, 4 and 7 days. As expected, the DDW group showed the lowest mineral content, whereas the CS-QP5 hydrogel group showed a significantly higher mineral content than the other groups on each day. With an increase in the inoculation time, the lesions became significantly shallower and the mineral content of the enamel surface layer increased in the CS-QP5 hydrogel, while the lesions of the QP5 and DDW groups became deeper and the mineral content decreased significantly. These results provided direct evidence of the remineralization promoted by the CS-QP5 hydrogel. These five sets of samples showed the greatest differences in the mineral content at a depth of 20–100 μm, whereas the mineral content in both the groups was similar at depths beyond 120 μm.

## Discussion

Dental caries is closely associated with the microbial metabolism of carbohydrates [42]. *Streptococcus mutans* has been identified as one of the main causative pathogens of caries because it can produce acid, synthesize extracellular polysaccharides and adhere to tooth surfaces [4]. This feed-forward imbalance in the microbial equilibrium leads to a continuous pH decline to the critical pH, below which tooth hard-tissue demineralization begins and dental caries gradually occurs. One critical factor contributing to biofilm inhibition or the remineralization of early enamel lesions is the retention time of the antimicrobial or remineralizing agents on the teeth. The traditional mouthwash raises a concern of the time interval between applications, which results in a reduced concentration of the agents over time between the doses. Inconsistent and insufficient application of the antimicrobial or remineralizing agents may allow cariogenic bacteria to recolonize on the tooth surface or lead to limited repair results of the early enamel lesions. Fluoride has a profound effect on caries prevention, for its qualities of cariostatic efficacy, inhibition of demineralization and promotion of incipient lesion remineralization [7, 8]. But it is far from a complete cure, and fluoride has the risk of fluorosis and black staining on tooth surface [9–11]. The chitosan hydrogel containing amelogenin-derived peptide (CS-QP5 hydrogel) may play a role of alternative, a new kind of effective non-fluoride anti-caries agent.

The antibacterial efficacy of the CS-QP5 hydrogel was evaluated using the CFU count, lactic acid production and the MTT assay of the biofilm metabolic activity. The bacterial biofilms were grown on the surface of the demineralized enamel blocks treated with different agents. With respect to the other groups, the *S. mutans* biofilm treated with the CS-QP5 hydrogel had a lower CFU count, lactic acid production, and metabolic activity even after seven days. Our results indicated that a sustainable retention of the hydrogel on the tooth surface provided a consistent amount of effective agents over a prolonged period of time, which may decrease the cariogenic property of the dental biofilm with fewer potential side effects. This inhibition effect of the hydrogel on the *S. mutans* biofilm may be attributed largely to the presence of chitosan, as its positive charge after protonation can interact with the bacterial cell wall causing bacterial aggregation and death, thus leading to a direct or indirect decrease in the biomass, lactic acid production, and metabolic activity of the biofilm [25, 43]. Chitosan can also form its own film that acts as an effective antibacterial barrier on the tooth surface because of its broad-spectrum antimicrobial activity [44].

The biomimetic reconstruction of the tooth enamel is a significant topic of study in materials science and dentistry as a novel approach to the prevention, restoration, and treatment of defective enamel. In this study, the CS-QP5 hydrogel showed promising results with respect to the remineralization of the early enamel lesions in a carious biofilm model, as indicated by the SMHR, PLM and TMR assays. Though the cracks shown in polarized light photographs were induced by cutting and polishing procedures to obtain 300 μm slices, the results present good remineralizing effects of CS-QP5 hydrogel to a certain extent. We speculated that in the CS-QP5 hydrogel, QP5 was the crucial factor controlling the oriented growth of the hydroxyapatite crystals, as observed *in vitro* and in the animal models of enamel caries. Chitosan, for its part, may act simply as a carrier of QP5, albeit one with antibacterial activity. The peptide binds to the surface of the enamel and spontaneously self-assembles into fibrillar scaffolds in response to specific environmental triggers, thereby providing a biomimetic scaffold capable of nucleating the hydroxyapatite formation. The QPX repeats and the hydrophilic C-terminal tail promote the adsorption of both calcium and phosphate on the demineralized enamel. Based on the, isoelectric point, the peptide may have multiple anionic surfaces that can interact with Ca^2+^ in different environments. However, compared with the previously reported remineralization results in the pH cycling model, QP5 showed a decrease in the surface hardness recovery and the mineral content of a lesion body with an increase in the inoculation time, as QP5 had no effects on limiting the growth of cariogenic bacteria and its residence time with an effective concentration staying on the tooth surface was relatively short when it was applied in the form of an aqueous solution.

The development of caries is associated with a continuous pH change in the plaque biofilm due to the accumulation of acid by-products from the metabolism of fermentable carbohydrates [1]. This gradual acidification may potentiate the ability of the CS-QP5 hydrogel to re-mineralize enamel. As the pH decreases, the positive hydrogen ions from the acid bind to the negative phosphate and hydroxyl ions from the enamel, leading to mineral loss [45, 46]. The amino groups of the chitosan present on the enamel surface may be able to capture these hydrogen ions from the acid, forming a positive protective layer to prevent the diffusion of these hydrogen ions to the mineral surface. At the same time, this positive layer may ensure an electrostatic interaction with QP5, preventing its loss into the bulk saliva. Protonated chitosan can also interact with the bacterial cell wall, causing bacterial aggregation and death. When the saliva restores the pH to the normal range of 6.3–7.0, chitosan can no longer interact with QP5, liberating the peptide to promote the remineralization of the enamel.

In this biofilm model, a dynamic balance was observed between the demineralization caused by carious bacteria and the remineralization due to the calcium and the phosphate present in the environment. When the effect of the demineralization was stronger than that of the remineralization, the mineral loss of the teeth started, resulting in the caries. Once the CS-QP5 hydrogel was applied, the remineralization effect was strengthened and exceeded the demineralization effect, thus promoting the repair of early enamel lesions. On the one hand, protonated chitosan interacted with the cell wall of the bacteria in the biofilms and inhibited the growth of the *S. mutans* biofilm, lactic acid production, and the metabolic activity, leading to a decrease in the demineralization. On the other hand, the peptide in the chitosan hydrogel captured the calcium and the phosphate from the environment and guided them to sequentially deposit on the surface of the demineralized enamel, thereby promoting the remineralization progress.

In summary, we have developed a CS-QP5 hydrogel with antibacterial effects as well as the remineralization effects of the initial enamel carious lesions even in a biofilm model over a prolonged period of time. However, we must acknowledge that the oral environment is complicated, as the temperature, pH, oral bacteria, and the volume and compositions of fluids fluctuate frequently. The antibacterial and the remineralization activity of the hydrogel need to be further optimized under more clinically relevant conditions.

## Conclusion

This chitosan hydrogel containing the amelogenin-derived peptide (CS-QP5 hydrogel) can inhibit an *S. mutans* biofilm and promote the remineralization of the initial enamel caries even in a biofilm model over a prolonged period of time. This hydrogen is promising as a brand-new anti-caries agent with various research prospects and practical application value.
